# Silyl-naphthalene endoperoxides as switchable sources of singlet oxygen for bactericidal activity†

**DOI:** 10.1039/d1ra02933a

**Published:** 2021-05-26

**Authors:** Min Qu, Nan Wu, Wanqing Jiang, Lei Wang, Mahinur S. Akkaya, Engin U. Akkaya

**Affiliations:** State Key Laboratory of Fine Chemicals, Dalian University of Technology 2 Linggong Road 116024 Dalian China eua@dlut.edu.cn; Department of Pharmaceutical Sciences, Dalian University of Technology 2 Linggong Road 116024 Dalian China leiwang@dlut.edu.cn; School of Bioengineering, Dalian University of Technology 2 Linggong Road 116024 Dalian China msa@dlut.edu.cn

## Abstract

Singlet oxygen is a short half-life cytotoxic agent which can be generated by chemical and photochemical methods. In order to make use of its antibacterial action at a selected location, it is desirable to have singlet oxygen in a relatively stable, “caged” structure, in the form of an endoperoxide. Here, the trimethylsilyl (TMS) group supplies the steric bulk, inhibiting the cycloreversion reaction to produce very little singlet oxygen under ambient conditions. However, when fluoride ions are added as tetrabutylammonium fluoride, very rapid removal of the TMS group takes place, followed by the unhindered cycloreversion, releasing singlet oxygen much faster. The bactericidal action on surfaces was demonstrated using *E. coli*, and imaged under fluorescence microscopy. Considering the issues related to emergence of antibiotic resistant bacterial strains, “on demand singlet oxygen” appears to be an exciting alternative.

## Abstract 2

Fluoride ions remove the bulky stopper, releasing singlet oxygen to kill harmful bacteria.

## Graphical abstract

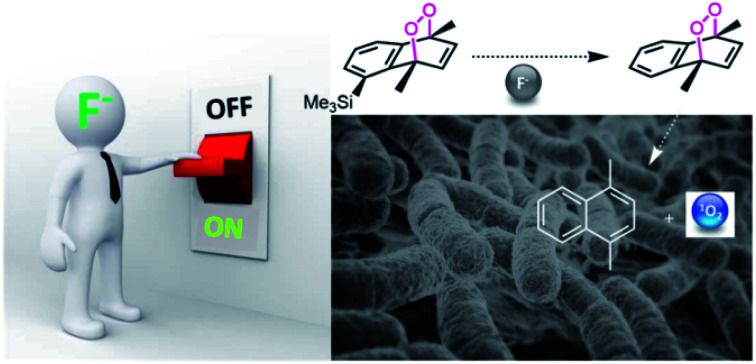

## Introduction

Among the reactive oxygen species (ROS), singlet oxygen is the most reactive and short-lived.^1^ In aqueous solutions, the half-life of singlet oxygen is about 3.5 microseconds.^2^ As a result of its reactivity, it can react with lipids, amino acids, nucleic acids and most of the cellular components.^3^ Non-specific reactivity demands a tight spatiotemporal control of its generation, if this reactivity is to be harnessed in a cytotoxic or antibacterial agent. In the last few decades, photodynamic antibacterial activity has attracted attention,^4^ since the bactericidal effect of singlet oxygen, is not subject to limitations of ordinary anti-bacterial agents, such as bacterial antibiotic resistance. It is also important to note that, ROS and specifically singlet oxygen is believed to be ultimate cause of bactericidal action of most antibiotics, if not all.^5^ We propose that the chemical (non-photonic) generation of singlet oxygen could extend the applicability of singlet oxygen as an effective antibacterial agent.

In recent years, we have provided evidence that singlet oxygen release reaction rate can be controlled by exogenic and endogenic (biological) modulators.^6^ Various naphthalene endoperoxide cycloreversion rates^7^ which were studied in more detail than other endoperoxide reactions,^8^ were shown by us^6*b*,*c*^ and others^9^ to be highly sensitive to steric crowding near the endoperoxide bridge. When sterically crowded, the singlet oxygen release rate is significantly slowed down. If this block can be removed by the modulators mentioned above in a fast process (chemical or photochemical) singlet oxygen release could be effectively controlled in both space and time.

## Experimental

### Materials and instrumentation

All commercial chemicals were used as supplied unless otherwise indicated. Anhydrous solvents were obtained from a Solvent Purification System. Column chromatography was performed using silica gel (200–300 mesh). ^1^H and ^13^C NMR spectra were recorded on Bruker Avance II 400 MHz or Bruker Avance III 500 MHz. Signal splitting patterns were described as singlet (s), doublet (d), triplet (t), quartet (q) and multiplet (m) with coupling constants (*J*) in hertz (Hz). High resolution mass spectra (HRMS) were recorded with an Agilent mass spectrometer. Reactions were monitored by thin-layer chromatography using Merck TLC Silica gel 60 F254.

#### Synthesis of compound 2

2-(2-Bromophenyl)-*N*-methoxy-*N*-methylacetamide: a mixture of 2-bromophenylacetic acid (1) (6.45 g, 30 mmol), EDC·HCl (6.9 g, 36 mmol), DMAP (0.37 g, 3 mmol), DIPEA (6.27 mL, 36 mmol), and *N*,*O*-dimethylhydroxylamine hydrochloride (3.51 g, 36 mmol) in dichloromethane (150 mL) was stirred at room temperature for 12 h. The reaction mixture was concentrated under vacuum, and the residue was diluted with ethyl acetate (200 mL). The organic layer was washed with saturated NaHCO_3_ (50 mL × 2) and brine (50 mL × 2), dried over Na_2_SO_4_, and concentrated under vacuum. The crude product was purified by column chromatography (15%, EtOAc in *n*-hexane) to afford 2-(2-bromophenyl)-*N*-methoxy-*N*-methylacetamide (2) as a colorless oil. Yield: 7.4 g (95%). ^1^H NMR (400 MHz, chloroform-d) *δ* 7.56 (d, *J* = 7.8 Hz, 1H), 7.31–7.22 (m, 2H), 7.16–7.08 (m, 1H), 3.93 (s, 2H), 3.70 (s, 3H), 3.22 (s, 3H); ^13^C NMR (100 MHz, CDCl_3_) *δ* 171.2, 135.1, 132.6, 131.5, 128.6, 127.5, 125.1, 61.3, 39.6, 32.3.

#### Synthesis of compound 3

2-(2-Bromophenyl)-*N*-methoxy-*N*-methylpropanamide: to a solution of 2-(2-bromophenyl)-*N*-methoxy-*N*-methylacetamide (2) (5.16 g, 20 mmol) in anhydrous THF (50 mL) was added NaHMDS (1.0 M in hexane, 30 mL, 30 mmol) at −78 °C *via* syringe under Ar atmosphere. After stirring for 30 min, MeI (4.3 g, 60 mmol) was added at the same temperature. The reaction mixture was stirred at −78 °C for 1 h and 0 °C for additional 1 h. The reaction mixture was quenched with saturated NH_4_Cl and extracted with ethyl acetate (50 mL × 3). The organic layer was washed with brine, dried over MgSO_4_, and concentrated under vacuum. The residue was purified by column chromatography (10–15%, EtOAc in *n*-hexane) to afford 2-(2-bromophenyl)-*N*-methoxy-*N*-methylpropanamide (3) as a colorless oil. Yield: 4.95 g (90%). ^1^H NMR (400 MHz, chloroform-d) *δ* 7.56 (dd, *J* = 8.0, 1.3 Hz, 1H), 7.33 (dd, *J* = 7.8, 1.8 Hz, 1H), 7.26 (td, *J* = 7.6, 1.3 Hz, 1H), 7.08 (td, *J* = 7.8, 1.6 Hz, 1H), 4.65–4.49 (m, 1H), 3.41 (s, 3H), 3.16 (s, 3H), 1.38 (d, *J* = 7.0 Hz, 3H); ^13^C NMR (100 MHz, CDCl_3_) *δ* 173.9, 140.4, 131.8, 127.2, 127.1, 126.9, 123.0, 60.0, 40.6, 31.4, 17.2.

#### Synthesis of compound 4

To a solution of 2-(2-bromophenyl)-*N*-methoxy-*N*-methylpropanamide (3) (4.9 g, 18 mmol) in dichloromethane (50 mL) was added a solution of DIBAL-H (1.0 M in cyclohexane, 27 mL, 27 mmol) at 78 °C *via* syringe under Ar atmosphere. The reaction solution was stirred at 78 °C for 1 h and quenched with 1.0 N HCl. The organic layer was washed with brine, dried over MgSO_4_, and concentrated under vacuum. The residue was purified by column chromatography (5% EtOAc in *n*-hexane) to afford 2-(2-bromophenyl)propanal (4) as a colorless oil. Yield: 3.07 g (81%). ^1^H NMR (400 MHz, chloroform-d) *δ* 9.74 (s, 1H), 7.64 (dd, *J* = 8.0, 1.3 Hz, 1H), 7.33 (td, *J* = 7.6, 1.3 Hz, 1H), 7.18 (td, *J* = 7.8, 1.7 Hz, 1H), 7.12 (dd, *J* = 7.7, 1.7 Hz, 1H), 4.17 (q, *J* = 7.1 Hz, 1H), 1.43 (d, *J* = 7.1 Hz, 3H); ^13^C NMR (100 MHz, CDCl_3_) *δ* 199.1, 136.8, 132.3, 128.2, 128.0, 127.1, 124.1, 50.9, 13.0.

#### Synthesis of compound 5

Propyne (0.52 g, 13 mmol) was placed in a dry argon-flushed, 200 mL round-bottomed flask equipped with a stirring bar and dissolved in dry CH_2_Cl_2_ (60 mL). Titanium tetrachloride (10 mmol, 10 mL of a 1.0 M CH_2_Cl_2_ solution) was added *via* a syringe at room temperature. Then, 2-(2-bromophenyl)propanal (4) (2.13 g, 10 mmol) was added *via* a syringe at room temperature. The reaction mixture was allowed to stir for 4 h and then was hydrolyzed with water. The mixture was extracted into hexanes, and the organic layer separated, dried over anhydrous MgSO_4_, concentrated under reduced pressure, and purified by flash column chromatography to afford 5-bromo-1,4-dimethylnaphthalene (5) as a colorless liquid. Yield: 1.1 g (47%). ^1^H NMR (400 MHz, chloroform-d) *δ* 7.88 (d, *J* = 8.4 Hz, 1H), 7.76 (d, *J* = 7.4 Hz, 1H), 7.15–7.19 (m, 2H), 7.12 (d, *J* = 7.2 Hz, 1H), 3.00 (s, 3H), 2.54 (s, 3H); ^13^C NMR (100 MHz, CDCl_3_) *δ* 135.6, 133.5, 133.3, 133.2, 131.5, 130.6, 127.1, 125.4, 124.9, 120.7, 26.4, 20.4.

#### Synthesis of compound 6

Magnesium turnings (0.17 g, 7 mmol) was activated with catalytic amount of iodine in 5 mL of THF for 15 minutes with vigorous stirring under Ar atmosphere. 5-Bromo-1,4-dimethylnaphthalene (5) (0.71 g, 3 mmol) was added to the reaction mixture in ice-bath. Then, TMS-Cl (0.38 g, 3.6 mmol) was dissolved in 3.0 mL THF and was added to the reaction mixture dropwise. The reaction mixture was allowed to stir at room temperature for 18 hours. Then, the mixture was extracted with water and DCM for three times. After removal of the solvent by rotary evaporator, the crude product was purified by silica gel column chromatography with hexane : EtOAc (95 : 5, v/v) as the eluent to afford (5,8-dimethylnaphthalen-1-yl)trimethylsilane (6) as a colorless oil. Yield: 0.3 g (44%). ^1^H NMR (400 MHz, chloroform-d) *δ* 7.95 (dd, *J* = 8.4, 1.1 Hz, 1H), 7.81 (dd, *J* = 6.9, 1.0 Hz, 1H), 7.36–7.31 (m, 1H), 7.22–7.06 (m, 2H), 2.75 (s, 3H), 2.58 (s, 3H), 0.38 (s, 9H); ^13^C NMR (100 MHz, CDCl_3_) *δ* 136.9, 136.1, 134.6, 132.58, 132.5, 132.0, 127.4, 125.5, 125.1, 122.7, 23.5, 19.1, 2.5.

#### Synthesis of compound 7

(5,8-dimethylnaphthalen-1-yl)trimethylsilane (6) (0.3 g, 1.32 mmol) was dissolved in 10 mL DCM. The reaction mixture was cooled to 0 °C in the ice bath. Methylene blue (0.04 g, 0.14 mmol) was added into the solution and mixture was stirred for 5 hours under oxygen atmosphere. During the reaction, 18 W, 630 nm red light was used. After removal of the solvent by rotary evaporator, the crude product was purified by silica gel column chromatography with hexane : EtOAc (95 : 5, v/v) as the eluent. The product 7 was obtained in colorless oil. Yield: 0.33 g (98%). ^1^H NMR (400 MHz, chloroform-d) *δ* 7.44 (dd, *J* = 7.8, 1.2 Hz, 1H), 7.29 (dd, *J* = 7.4, 1.2 Hz, 1H), 7.19–7.11 (m, 1H), 6.61 (s, 2H), 1.96 (s, 3H), 1.78 (s, 3H), 0.35 (s, 9H) ;^13^C NMR (100 MHz, CDCl_3_) *δ* 147.1, 140.0, 139.2, 138.6, 133.2, 131.5, 124.6, 120.7, 80.4, 77.0, 18.5, 15.7, 1.9.

## Results and discussion

**Fig. 1:**
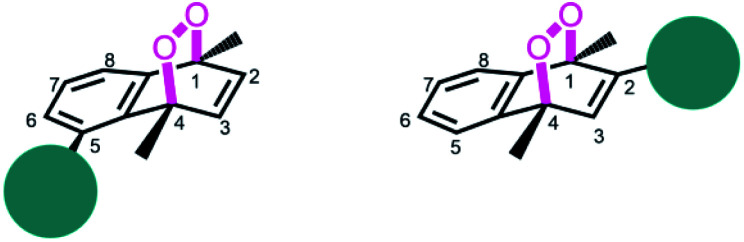
Bulky substituents near the endoperoxide bridge is known to inhibit cycloreversion reaction rate.

**Fig. 2:**
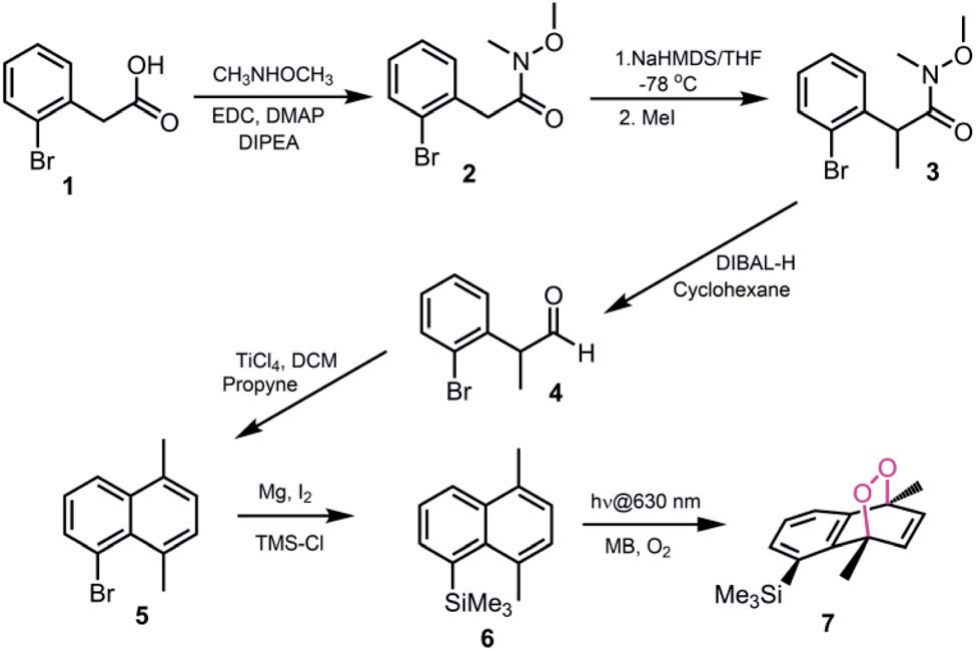
The synthesis of 5-TMS-1,4-dimethylnaphthalene 6 and its corresponding endoperoxide 7.

Based on the reported effects of 5-methyl substitution (Fig. 1) on the cycloreversion rate of 1,4-dimethylnaphthalene endoperoxide (1,4),^10^ we targeted the compound 7 for synthesis. Previously, we synthesized its structural isomer where the steric hindrance is on C-2, but steric block on the other side of the bridgehead substitution, may offer additional advantages in deprotection rates and/or stability of the initial endoperoxide. The synthesis (Fig. 2) of compound 7 makes use of an effective methodology^10^ which allows the preparation of substituted naphthalenes, starting from substituted phenylacetaldehyde (4, in this case) and the appropriate alkyne. Aldehyde itself can be obtained from the commercially available materials in just two steps. Once the naphthalene core 5 is constructed, the bulky trimethylsilyl (TMS) group is substituted *via* a Grignard reaction. Irradiation of compound 6 under oxygen atmosphere with methylene blue (MB) as a photosensitizer, and a red LED array emitting at 630 nm, gives the endoperoxide compound in high yields. Endoperoxide 7 can be separated from the reactant and any unreacted material from the previous step (compound 5) by silica gel column chromatography.

**Fig. 3:**
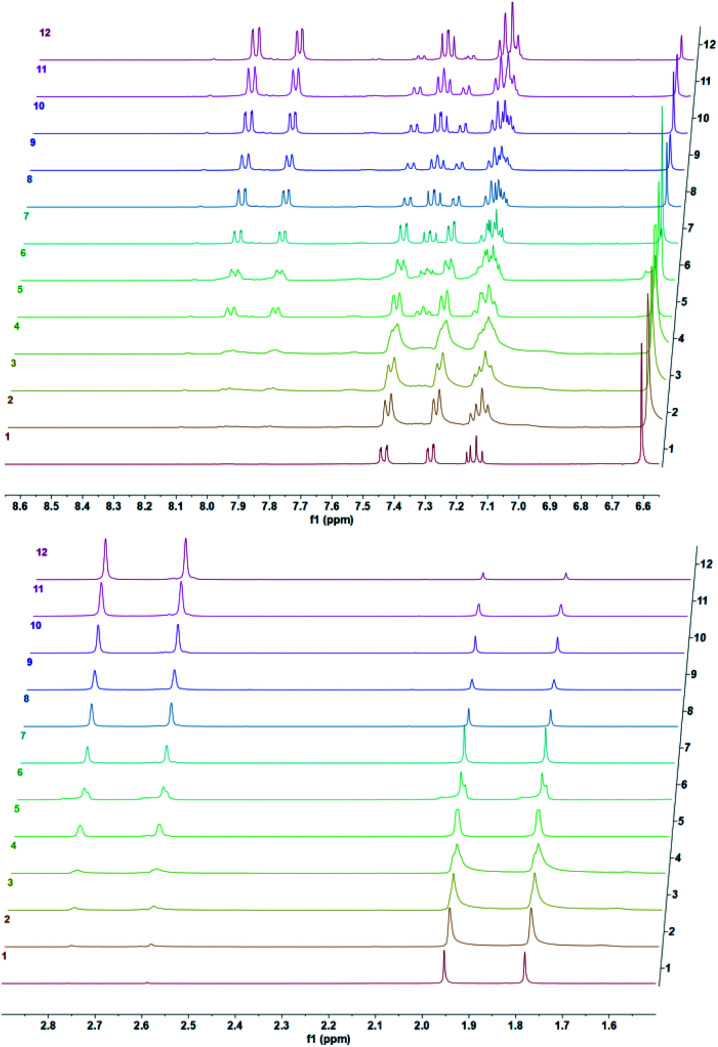
Temporal evolution of ^1^H NMR spectra of the endoperoxide 7 in CDCl_3_ at 37 °C. Top downfield region, bottom: upfield region of the spectra. From bottom to top: 0 h, 0.5 h, 1.5 h, 2.5 h, 5.5 h, 8 h, 10 h, 12 h, 24 h, 33 h, 52 h, 73 h.

We studied cycloreversion rate of the compound 7 by ^1^H NMR (Fig. 3). As expected, the TMS substituent at the 5 position slowed down the cycloreversion reaction. We were able to determine the half-life (ESI) as 125 h at 25 °C. Compared to the unsubstituted 1,4-dimethylnaphthalene endoperoxide 11, the cycloreversion reaction rate is 25 times slower.

**Fig. 4:**
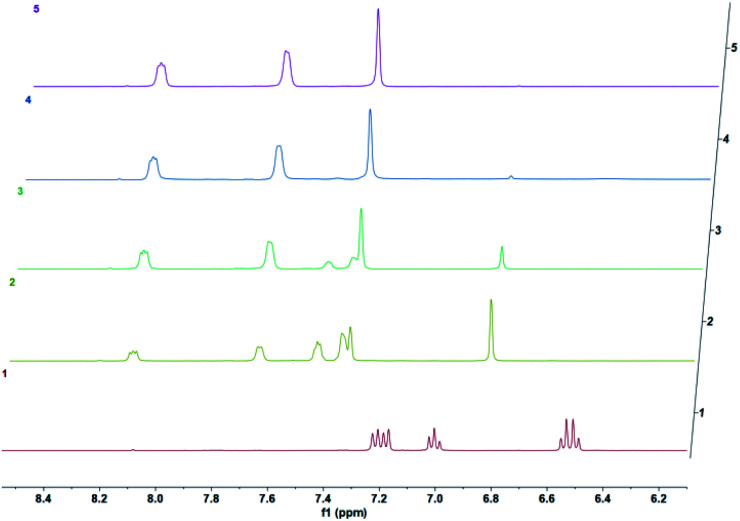
Temporal evolution of ^1^H NMR spectra of the endoperoxide 7 in DMSO-*d*_6_ following the addition of TBAF after *t* = 0 at 37 °C in DMSO-*d*_6_ as the solvent. 0 h, 5 min, 1.5 h, 3.5 h, 5 h. The conversion of 7 to 9 is complete in 5 minutes under these conditions.

**Fig. 5:**
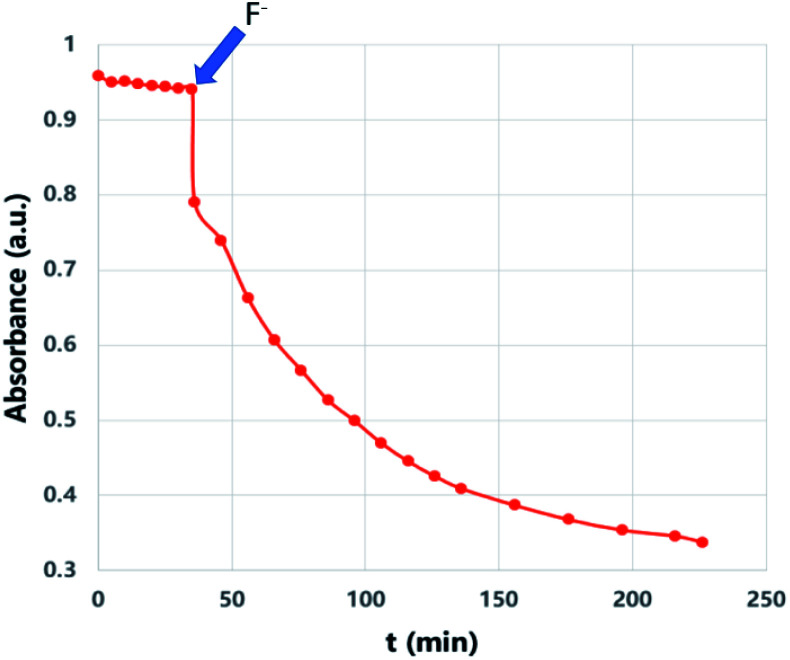
“Off–On” switching of ^1^O_2_ release: when fluoride (6 mM) in the form of TBAF in THF was added, the trap (DPBF, 27 μM) absorbance in DMSO at 414 nm starts to decrease sharply in a pseudo-first order reaction, coupled to the cycloreversion of endoperoxide 7 (200 μM).

**Fig. 6:**
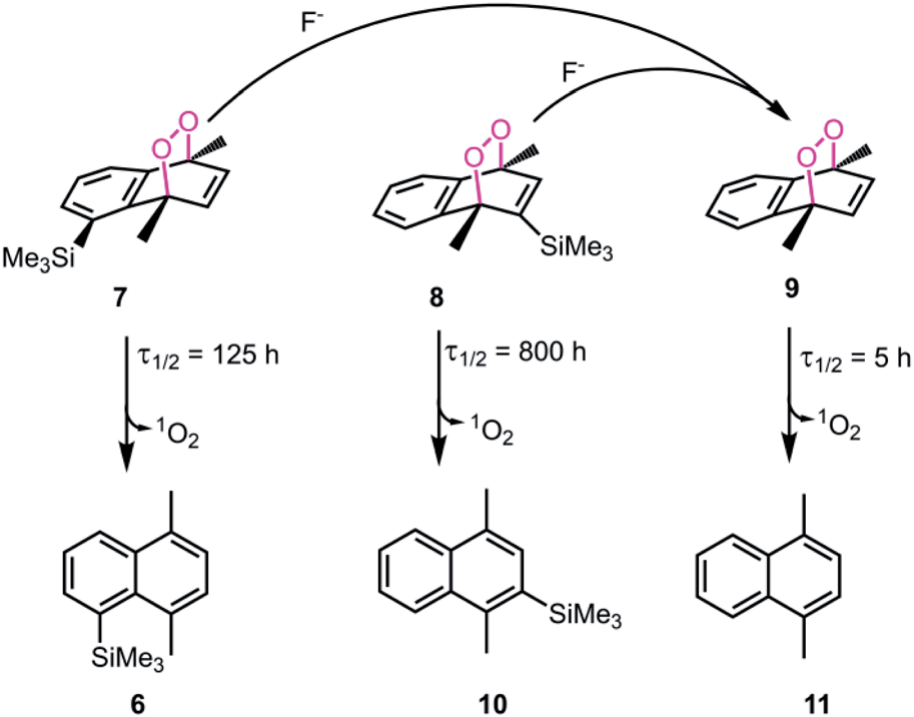
Steric inhibition of cycloreversion as illustrated with compound 7 and selected endoperoxide derivatives from literature.^6^ The cycloreversion rates of 1,4-dimethylnaphthalene endoperoxides were determined at 25 °C. Steric bulk at positions 2 and 5 seem to be a significant stabilizing factor for the endoperoxide. The other product formed in all of the cycloreversion reactions is singlet oxygen (^1^O_2_).

We then wanted to demonstrate that the addition of fluoride indeed results in a fast removal of the TMS-steric inhibitor group, which is followed by an accelerated cycloreversion. The ^1^H NMR data shown in Fig. 4 clearly show these changes. The first (*t* = 0) NMR shows the typical protons of the endoperoxide 7 in the 8.4–6.2 ppm region. Immediately after the addition of TBAF in THF, TMS is removed completely within 5 min (spectrum 2). ^1^H NMR data confirm the fact that removal of the TMS group is much faster than the cycloreversion reaction of the endoperoxide 7. Then the transformation of 9 to 11 takes place at a much faster rate. In order to confirm the identity and rate of the other product of cycloreversion, a solution of the endoperoxide 7 was prepared in DMSO, and the singlet oxygen probe 1,3-diphenyl-iso-benzofuran (DPBF) was added. As the reaction proceeds, cycloaddition of singlet oxygen initiates degradation of the benzofuran structure, and the absorbance peak at 414 nm due the benzofuran decreases (Fig. 5) as the singlet oxygen is produced. The reaction is highly specific to singlet oxygen. Clearly, fluoride addition causes a much accelerated release of singlet oxygen, which is due to the rapid “uncaging” of the endoperoxide. The general scheme of TMS-removal coupled acceleration of singlet oxygen release is presented in Fig. 6. Finally, we wanted to demonstrate the antibacterial action of singlet oxygen generated by fluoride addition. For bacterial overnight culture, a single colony of *E. coli* (Dh5α) used to inoculate 2.0 mL LB for 18 h at 37 °C for constant shaking at 200 rpm. These cells were aliquoted into fresh tubes for endoperoxide/F^−^ and mock treatments. Varying concentrations (40 mM) of endoperoxides 7 or 8 in DMSO and TBAF (100 mM) in DMSO were mixed at 1 : 1 (5 μM/5 μM) ratio, incubated for 30 min at RT for activation. Then 10 μL of the reaction mixture was added directly into fresh tubes, onto which the cells in 90 μL were added, and incubated for 30 minutes. The treated and mock treated cells were collected by spinning and washed with 200 μL phosphate-buffered saline (PBS) for two times. The cells were suspended and incubated in 20 μL acridine orange/ethidium bromide staining solution (prepared according to the manufacturer's instructions; BBI Life Sciences, Shanghai, PRC) in dark. The cells were collected by spinning and washed and suspended in 20 μL 1× buffer of the staining kit. The samples were investigated under bright field, FTIC, and TxRED filters using fluorescent microscope. The pictures were taken with exposures of 100 milliseconds for brightfield and 500 milliseconds exposures with FITC and TxRED filters at 100× magnification. We compared the bactericidal activity of the isomeric endoperoxides 7 and 8 at 2.0 mM concentration.

**Fig. 7:**
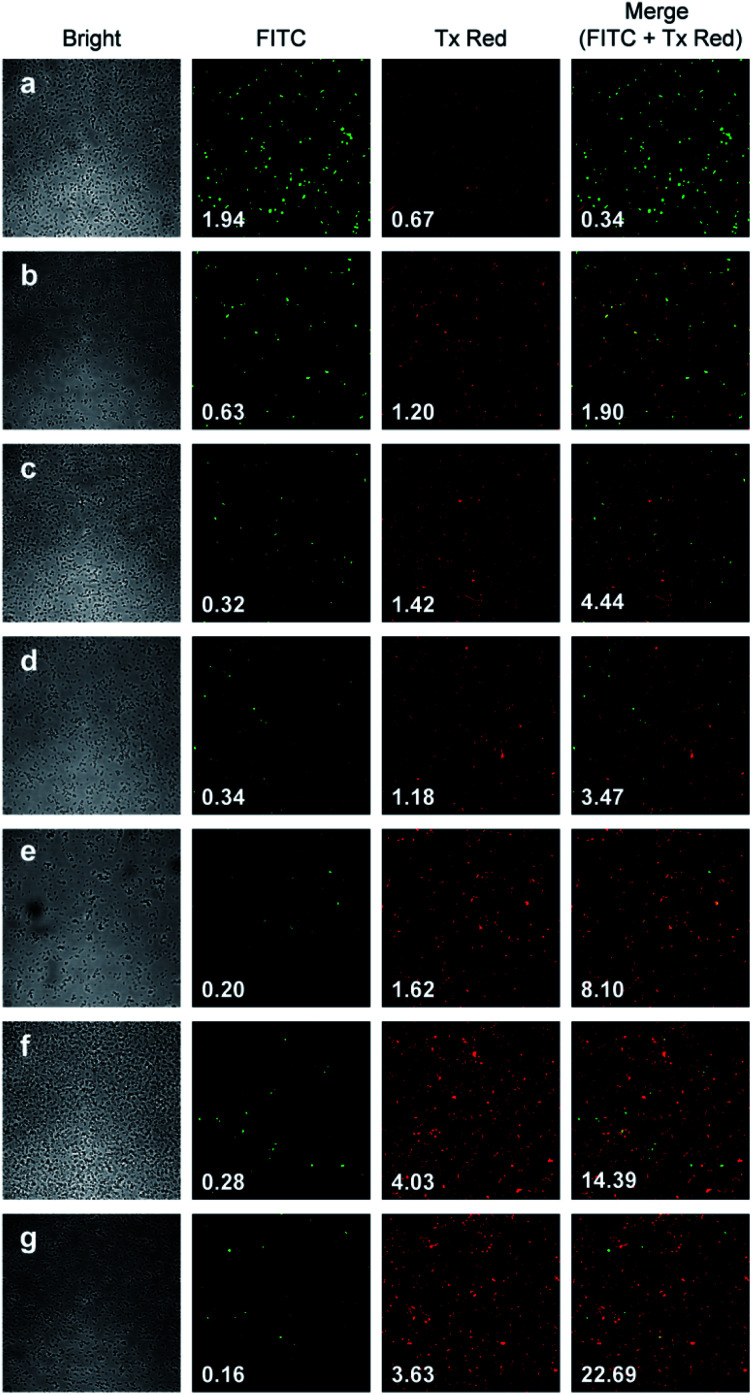
Dead/live assay picture matrix of the action of endoperoxides 7 or 8 activated by TBAF on *E. coli*. Red emission intensity to green emission intensity ratios are indicated in each picture at the bottom left. These are the resulting after 30 minutes incubation followed by washing and staining. (a) control, (b) 10% v/v DMSO, (c) 10% v/v DMSO, 5 mM TBAF; (d) 10% v/v DMSO, 2 mM endoperoxide 8; (e) 10% v/v DMSO, 2 mM endoperoxide 7; (f) 10% v/v DMSO, 2 mM endoperoxide 8, 5 mM TBAF; (g) 10% v/v DMSO, 2 mM endoperoxide 7, 5 mM TBAF. Column 1 from left: bright field images, Column 2: FITC filter, Column 3: TxRED filter, Column 4: merged. The numbers on the bottom left are total intensity in that filter range, except for the final column where it is the red to green ratio.

The ratio of the red channel total intensity (corresponding to dead cells) and the green channel total intensity (corresponding to live cells) is provided on the microscopy images (Fig. 7.). The microscopy data show that singlet oxygen generated by the action of fluoride on endoperoxide 7 has a demonstrable bactericidal effect, which significantly larger than endoperoxide 7 alone, and endoperoxide 8 + fluoride. The latter difference may be due to rate of TMS displacement.

## Conclusions

In summary, we present a proof of principle for the use of caged singlet oxygen compounds as potential bactericidal agents activated by fluoride ions. The current work, while presenting a novel and interesting synthesis of a 5-TMS-substituted naphthalene endoperoxide, it also demonstrates the generality of steric hindrance *via* silyl functionalization. We provide a stable endoperoxide which could be transformed into an effective antibacterial agent on exposure to fluoride for 5 minutes. These properties of the endoperoxide compounds could make them potentially useful especially in dental anti-bacterial applications. Work in that direction is currently in progress in our laboratories.

## Conflicts of interest

There are no conflicts to declare.

## Supplementary Material

RA-011-D1RA02933A-s001
