# Supplementary material for: Silyl-naphthalene endoperoxides as switchable sources of singlet oxygen for bactericidal activity
Source: RSC Adv. 2021 May 26;11(31):19083–7. doi: 10.1039/d1ra02933a (PMC9033937; doi:10.1039/d1ra02933a)
Supplement: RA-011-D1RA02933A-s001 [file RA-011-D1RA02933A-s001.pdf]

Electronic Supplementary Material (ESI) for RSC Advances.
This journal is © The Royal Society of Chemistry 2021

Electronic Supplementary Information

Silyl-naphthalene endoperoxides as switchable sources of singlet oxygen for bactericidal activity

Min Qu, Nan Wu, Wanqing Jiang, Lei Wang,* Mahinur S. Akkaya* and Engin U. Akkaya*

Table of Contents

NMR spectra S2 – S7

Reaction rate experiments S8 – S9

Singlet oxygen trap experiments S10

NMR spectra

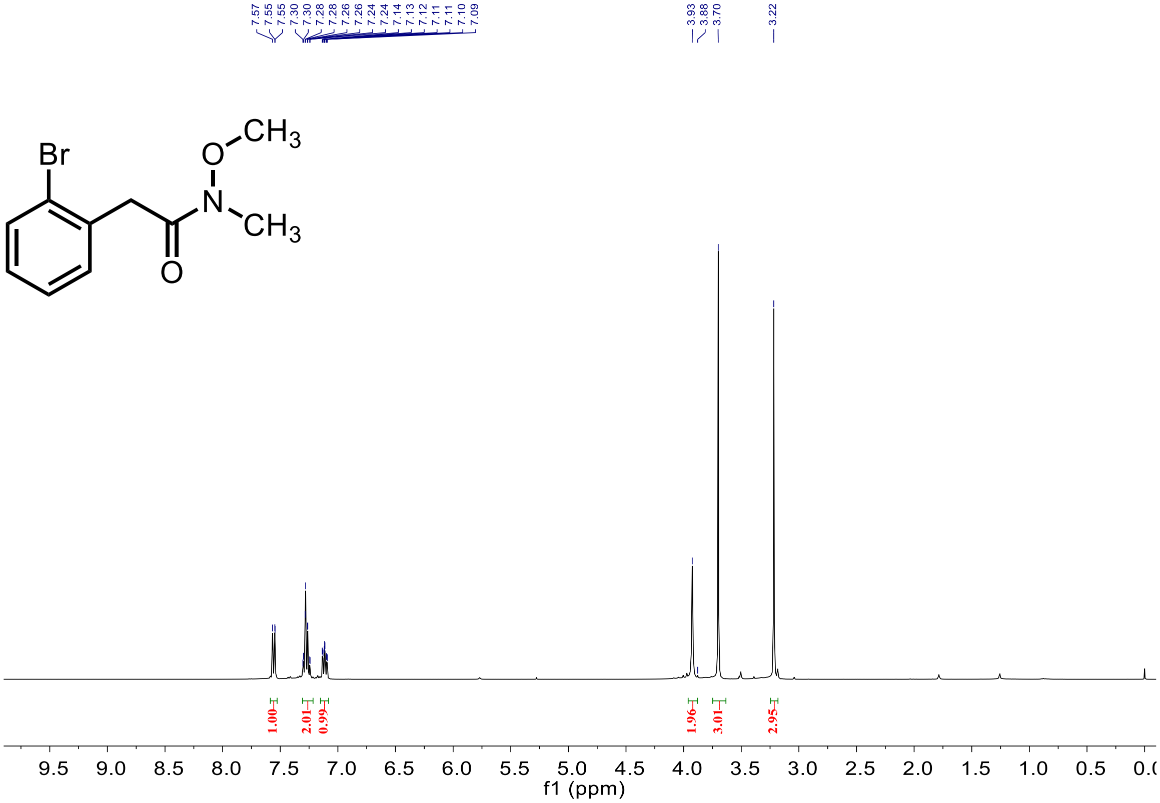
7.57
7.55
7.55
7.30
7.30
7.28
7.28
7.26
7.26
7.24
7.24
7.14
7.13
7.12
7.11
7.11
7.10
7.09
3.93
3.88
3.70
3.22
Br
O
CH3
N
CH3
O
1.00
2.01
0.99
1.96
3.01
2.95
f1 (ppm)

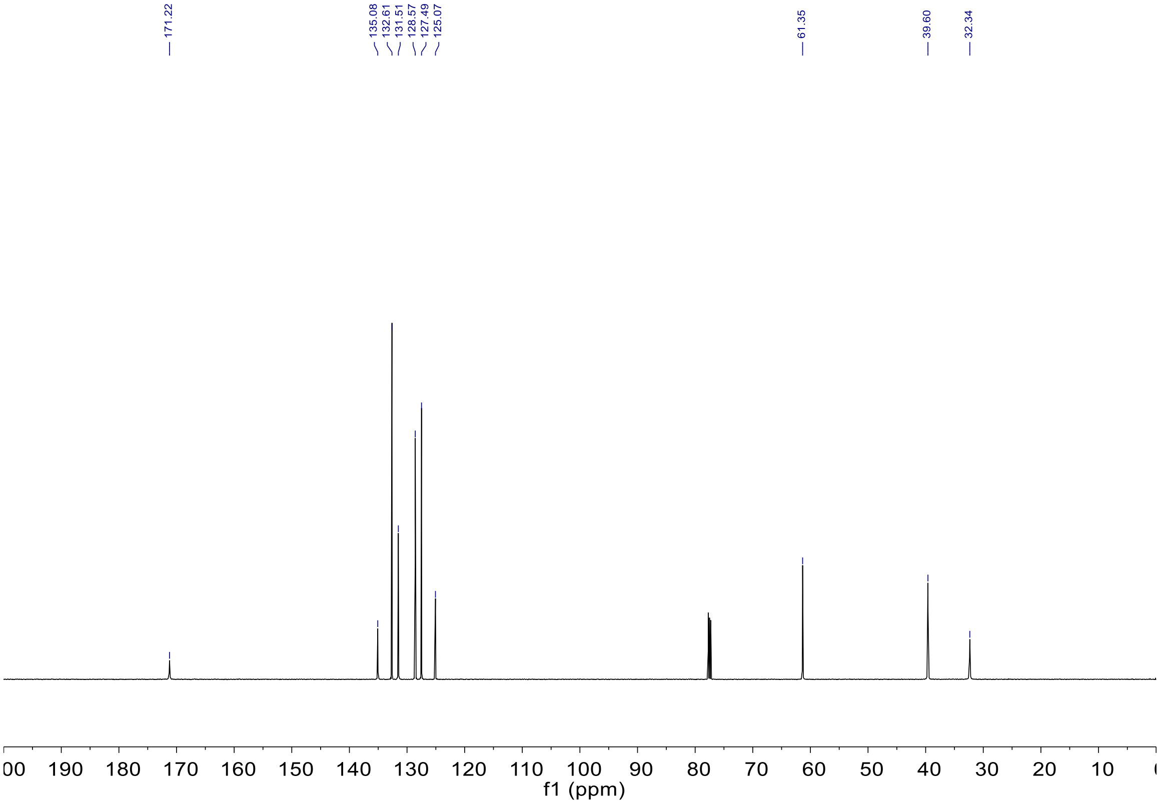
171.22
135.08
132.61
131.51
128.57
127.49
125.07
61.35
39.60
32.34
f1 (ppm)

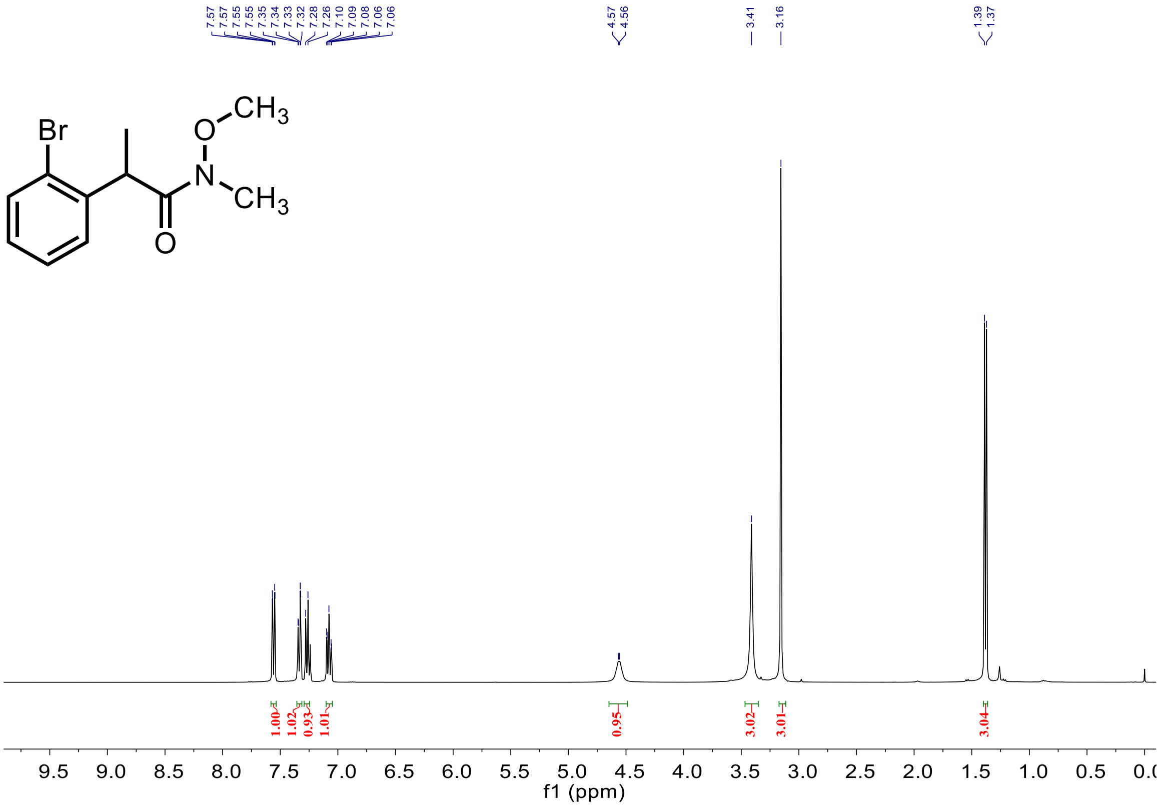
Br
O
CH3
N
CH3
O
7.57
7.57
7.55
7.55
7.35
7.34
7.33
7.32
7.28
7.26
7.10
7.09
7.08
7.06
7.06
4.57
4.56
3.41
3.16
1.39
1.37
1.00
1.02
0.93
1.01
0.95
3.02
3.01
3.04
f1 (ppm)

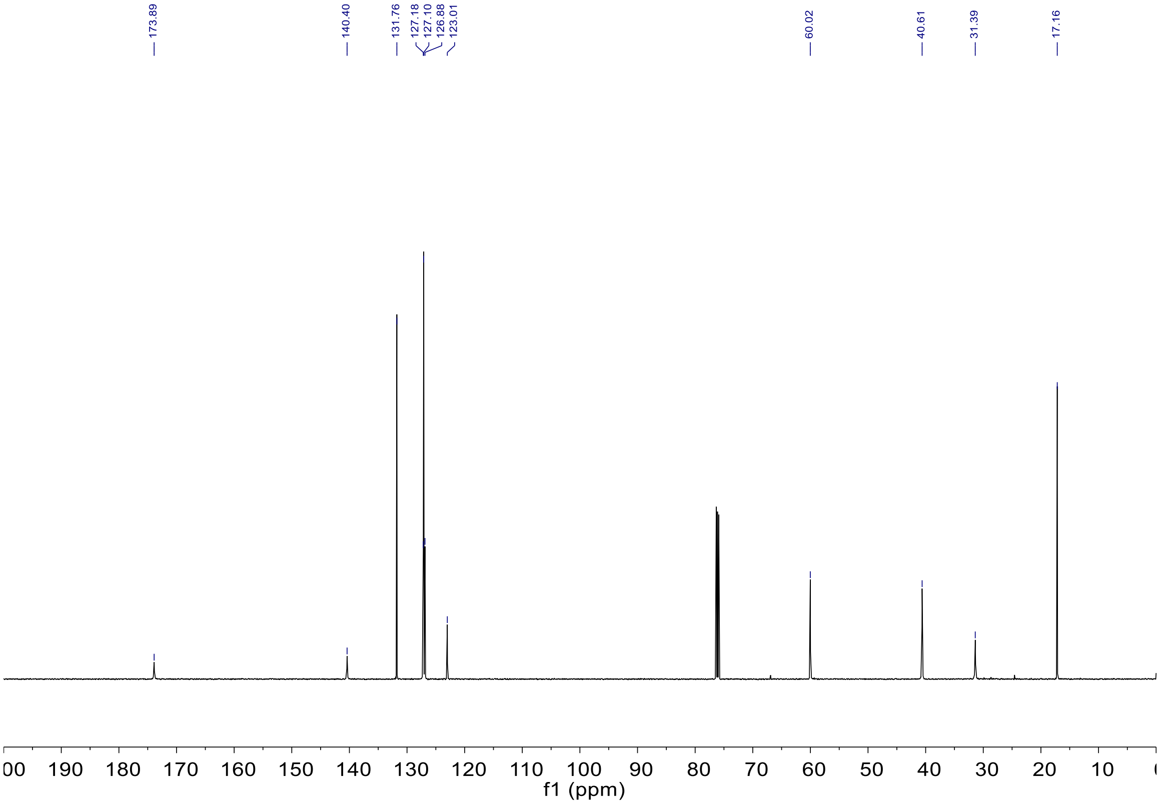
173.89
140.40
131.76
127.18
127.10
126.88
123.01
60.02
40.61
31.39
17.16
f1 (ppm)

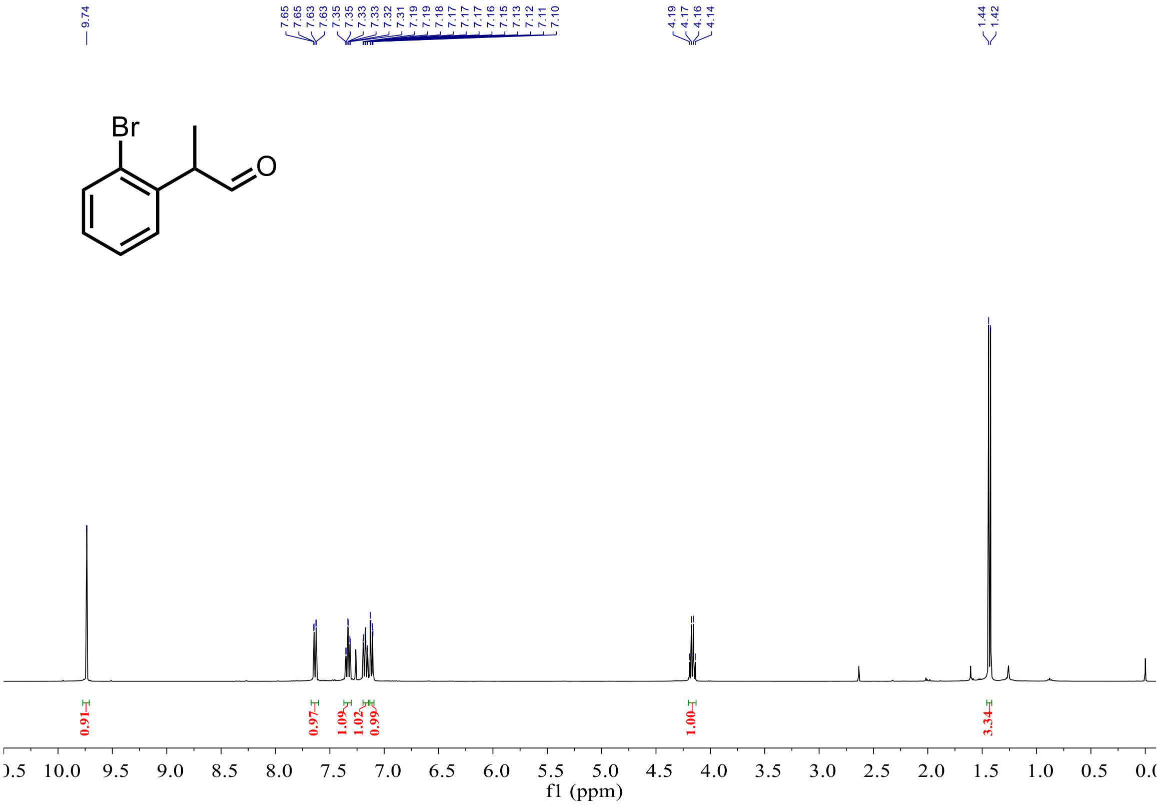
9.74
7.65 7.65 7.63 7.63 7.35 7.35 7.33 7.33 7.32 7.31 7.19 7.19 7.18 7.17 7.17 7.17 7.16 7.15 7.13 7.12 7.11 7.10
4.19 4.17 4.16 4.14
1.44 1.42
Br
O
0.91
0.97
1.09
1.02
0.99
1.00
3.34
f1 (ppm)

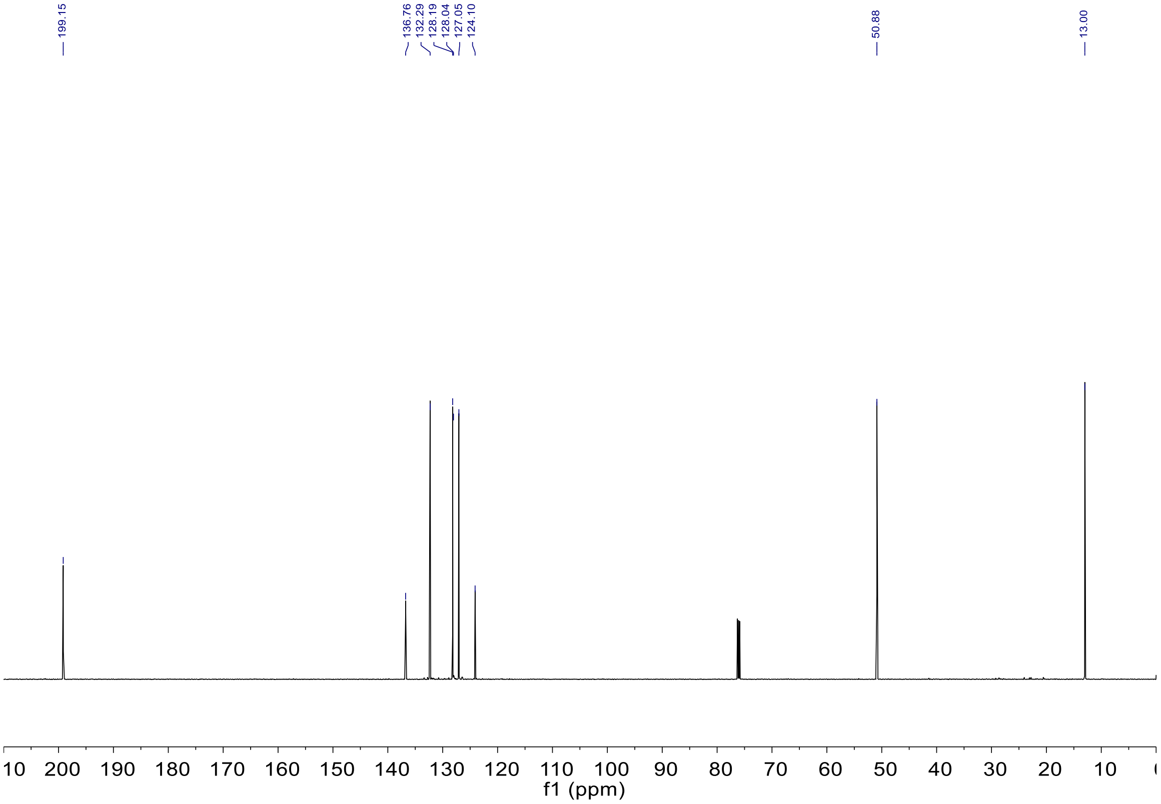
199.15
136.76 132.29 128.19 128.04 127.05 124.10
50.88
13.00
f1 (ppm)

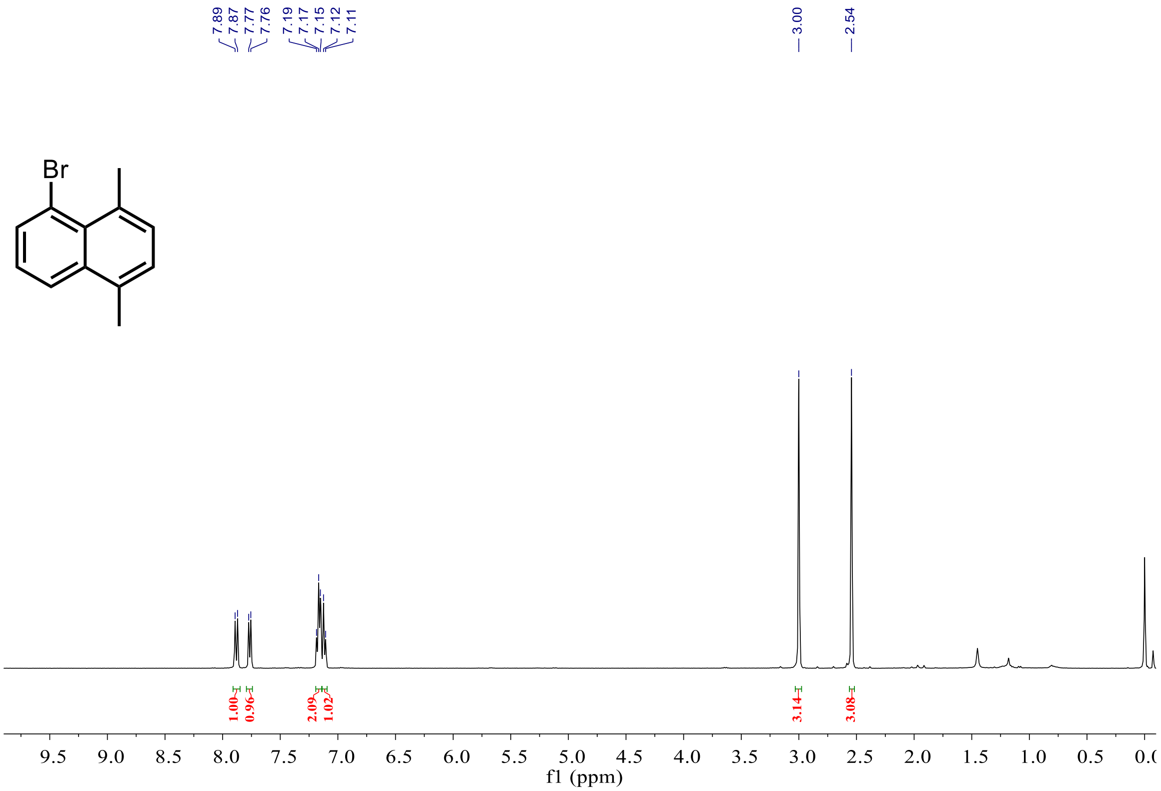
Br
7.89
7.87
7.77
7.76
7.19
7.17
7.15
7.12
7.11
3.00
2.54
1.00
0.96
2.09
1.02
3.14
3.08
9.5 9.0 8.5 8.0 7.5 7.0 6.5 6.0 5.5 5.0 4.5 4.0 3.5 3.0 2.5 2.0 1.5 1.0 0.5
f1 (ppm)

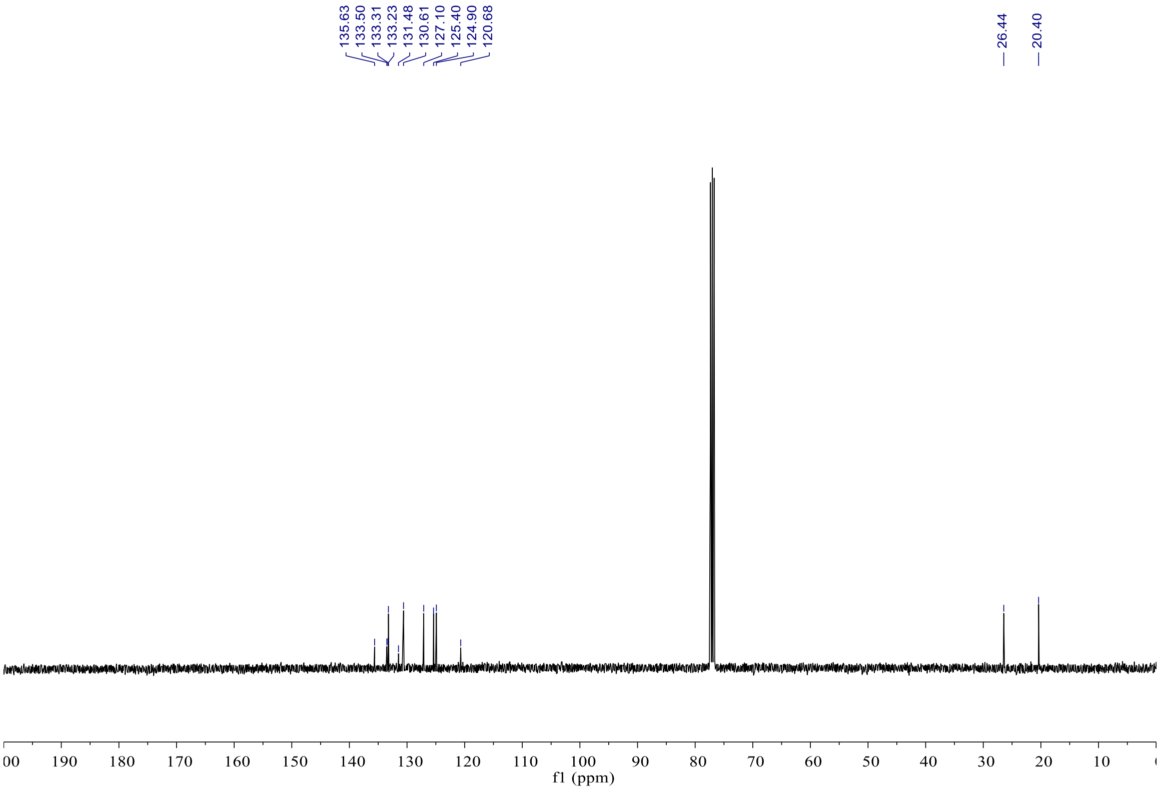
135.63
133.50
133.31
133.23
131.48
130.61
127.10
125.40
124.90
120.68
26.44
20.40
190 180 170 160 150 140 130 120 110 100 90 80 70 60 50 40 30 20 10
f1 (ppm)

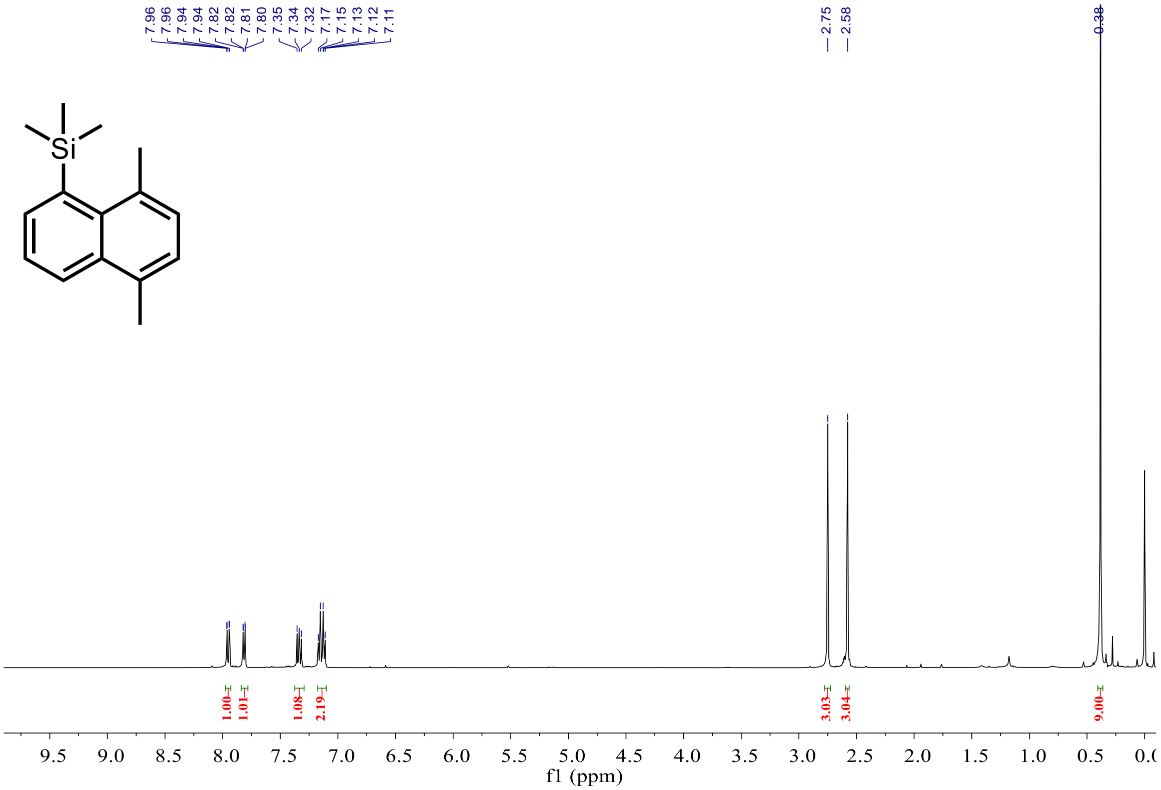
Si
7.96
7.96
7.94
7.94
7.82
7.82
7.81
7.80
7.35
7.34
7.32
7.17
7.15
7.13
7.12
7.11
2.75
2.58
0.38
1.00
1.01
1.08
2.19
3.03
3.04
9.00
9.5 9.0 8.5 8.0 7.5 7.0 6.5 6.0 5.5 5.0 4.5 4.0 3.5 3.0 2.5 2.0 1.5 1.0 0.5
f1 (ppm)

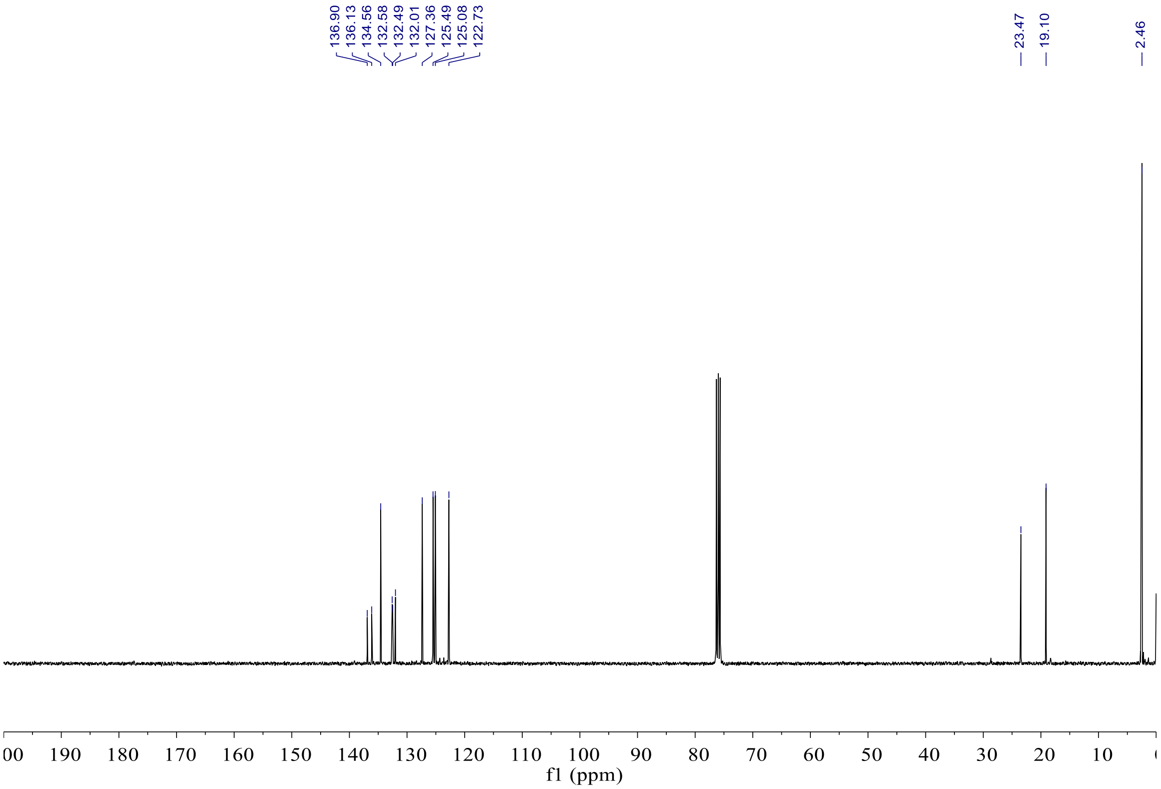
136.90
136.13
134.56
132.58
132.49
132.01
127.36
125.49
125.08
122.73
23.47
19.10
2.46
190 180 170 160 150 140 130 120 110 100 90 80 70 60 50 40 30 20 10
f1 (ppm)

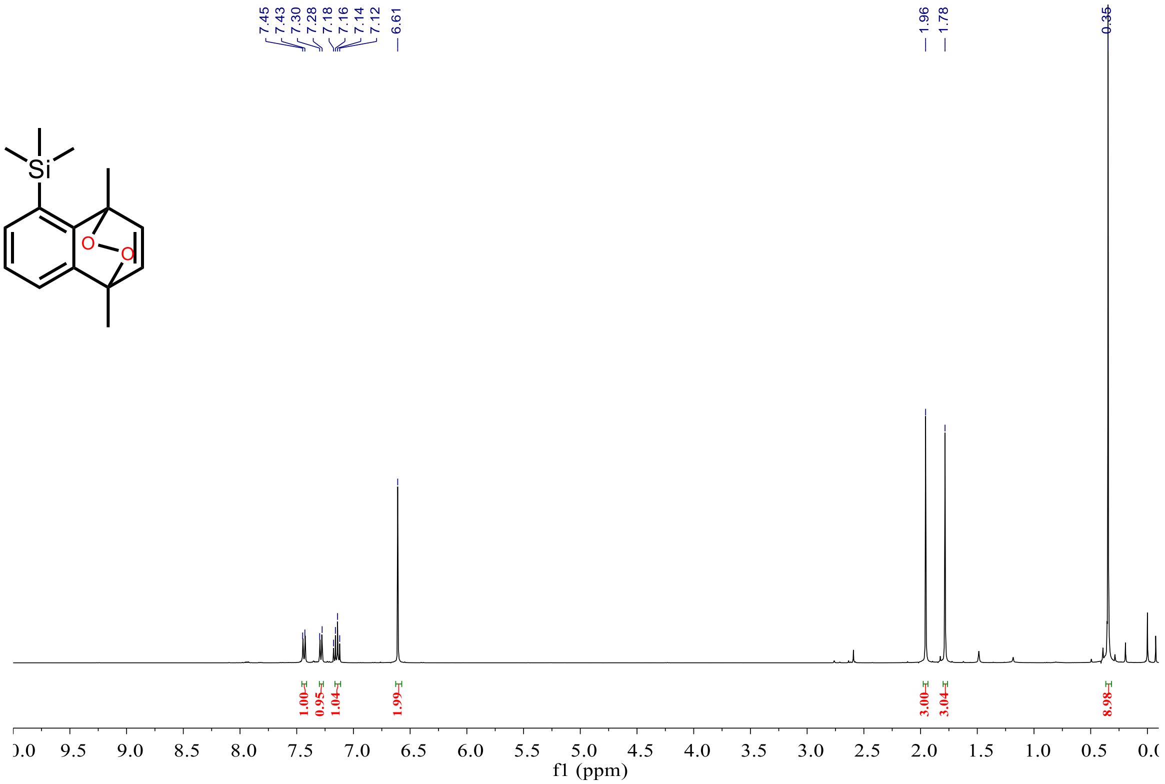
Si
O
O
7.45
7.43
7.30
7.28
7.18
7.16
7.14
7.12
6.61
1.96
1.78
0.35
1.00
0.95
1.04
1.99
3.00
3.04
8.98
f1 (ppm)

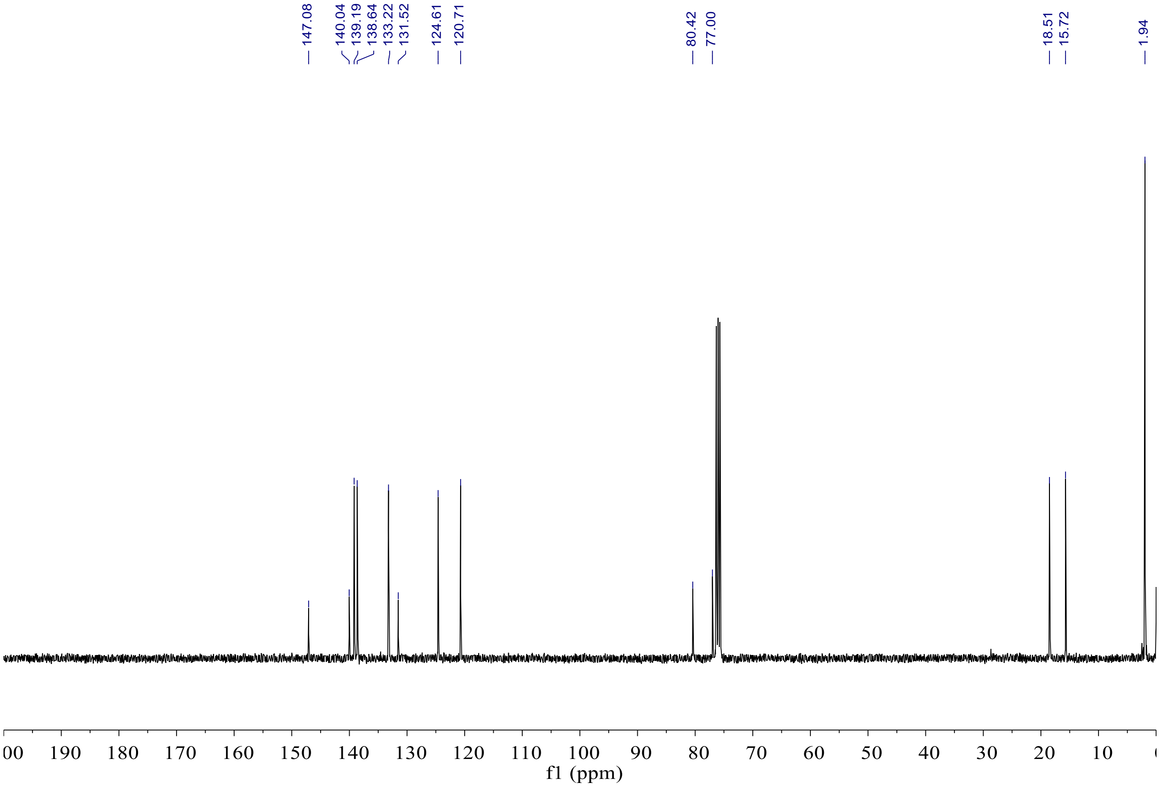
147.08
140.04
139.19
138.64
133.22
131.52
124.61
120.71
80.42
77.00
18.51
15.72
1.94
f1 (ppm)

Reaction rate experiments

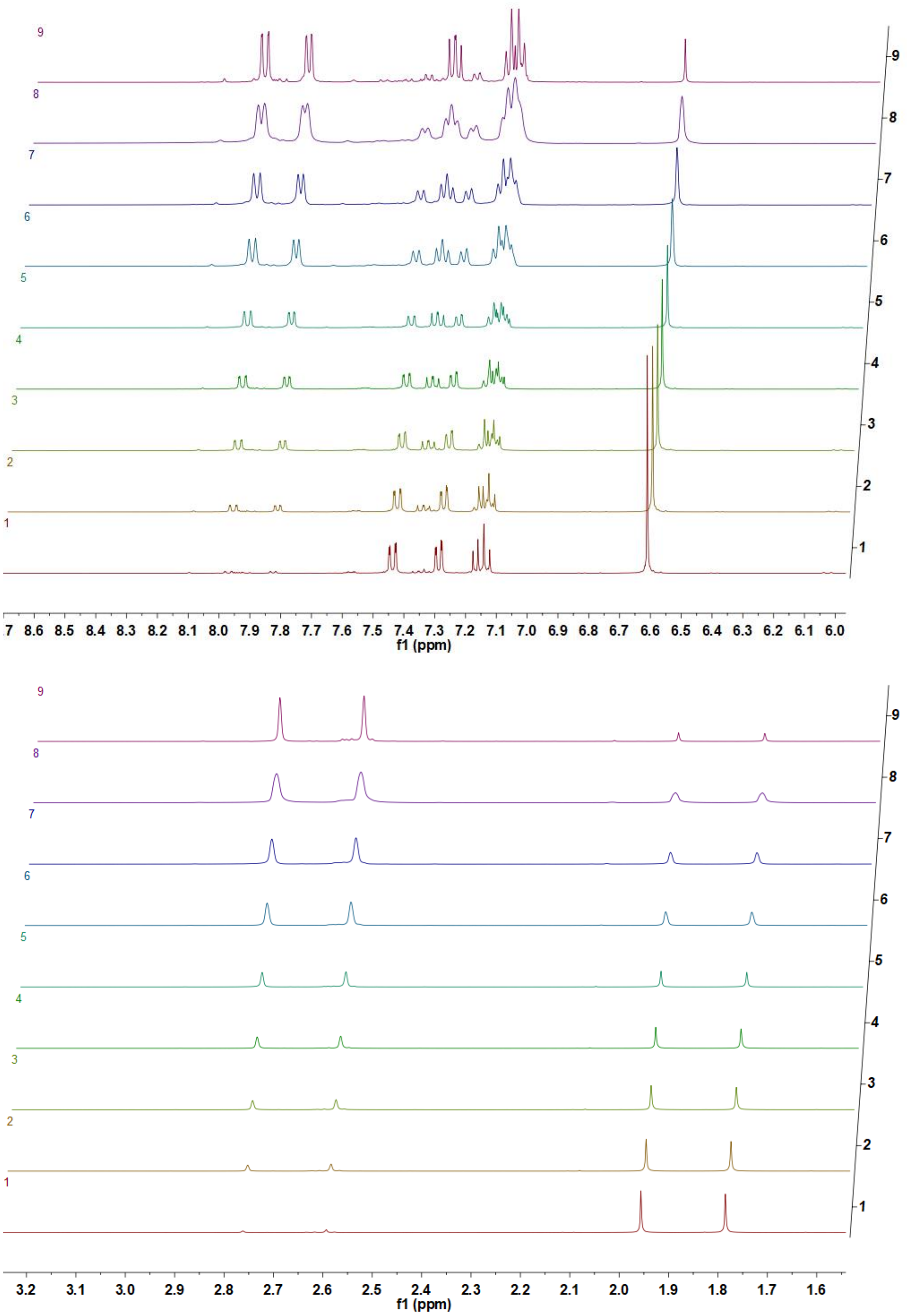

Figure S1. Evolution of the NMR spectra of endoperoxide with time at 25 ℃ in Chloroform-*d* as the solvent. (Spectra 1: 0 hours, Spectra 2: 30 hours, Spectra 3: 54 hours, Spectra 4: 77 hours, Spectra 5: 125 hours, Spectra 6: 173 hours, Spectra 7: 197 hours, Spectra 8: 246 hours, Spectra 9: 292 hours).

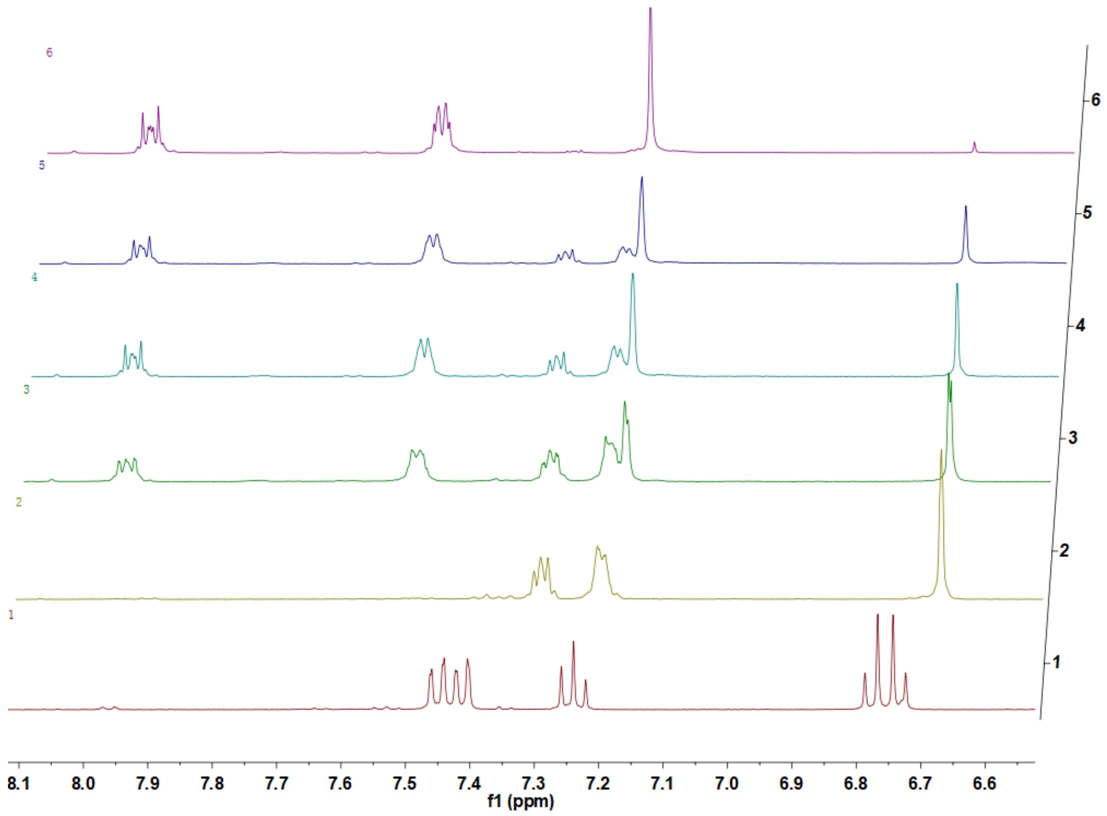

Figure S2. Evolution of the NMR spectra of TBAF added endoperoxide with time at 25 °C in DMSO-d_6 as the solvent. (Spectra 1: 0 hours, Spectra 2: 5 minutes, Spectra 3: 2 hours, Spectra 4: 3.5 hours, Spectra 5: 4.5 hours, Spectra 6: 16.5 hours).

Singlet oxygen trap experiments

In singlet oxygen generation experiments, 1,3-Diphenylisobenzofuran (DPBF) was used as chemical singlet oxygen trap molecule in DMSO. This procedure includes approximately 200 µM Compound **7** mixed with trap compound (approximately 27 µM) in DMSO. TBAF (approximately 6 mM) was added. Measurements were taken at 10 minutes intervals at 37 °C in dark conditions. Absorbance decrease of trap molecules at 414 nm was monitored, revealing singlet oxygen generation as a result of the silyl group removal.

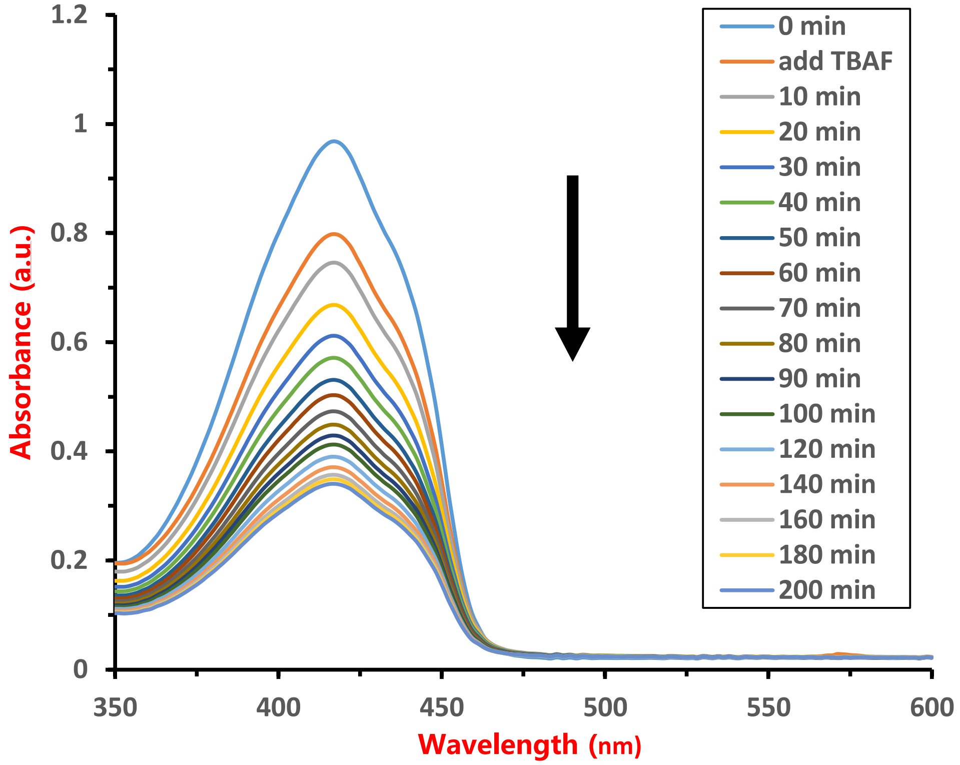

Figure S3. Decreasing absorbance peak for the singlet oxygen trap DPBF at 414 nm with time in DMSO in response to the addition of fluoride (TBAF) to compound **7.**
